# Coding stimulus amplitude by correlated neural activity

**DOI:** 10.1103/PhysRevE.91.042717

**Published:** 2015-04-28

**Authors:** Michael G. Metzen, Oscar Ávila-Åkerberg, Maurice J. Chacron

**Affiliations:** 1 Department of Physiology, McGill University, 3655 Sir William Osler, Montréal, Québec H3G 1Y6, Canada; 2 Department of Physics, McGill University, 3655 Sir William Osler, Montréal, Québec H3G 1Y6, Canada

## Abstract

While correlated activity is observed ubiquitously in the brain, its role in neural coding has remained controversial. Recent experimental results have demonstrated that correlated but not single-neuron activity can encode the detailed time course of the instantaneous amplitude (i.e., envelope) of a stimulus. These have furthermore demonstrated that such coding required and was optimal for a nonzero level of neural variability. However, a theoretical understanding of these results is still lacking. Here we provide a comprehensive theoretical framework explaining these experimental findings. Specifically, we use linear response theory to derive an expression relating the correlation coefficient to the instantaneous stimulus amplitude, which takes into account key single-neuron properties such as firing rate and variability as quantified by the coefficient of variation. The theoretical prediction was in excellent agreement with numerical simulations of various integrate-and-fire type neuron models for various parameter values. Further, we demonstrate a form of stochastic resonance as optimal coding of stimulus variance by correlated activity occurs for a nonzero value of noise intensity. Thus, our results provide a theoretical explanation of the phenomenon by which correlated but not single-neuron activity can code for stimulus amplitude and how key single-neuron properties such as firing rate and variability influence such coding. Correlation coding by correlated but not single-neuron activity is thus predicted to be a ubiquitous feature of sensory processing for neurons responding to weak input.

## I. INTRODUCTION

Correlated neural activity is observed ubiquitously in the brain [1,2] but its role in information coding remains controversial. Indeed, it has been argued that correlations can introduce redundancy in the neural code, thereby limiting information transmission [3,4]. However, more recent studies have challenged this notion and have instead argued that correlated neural activity could carry information independently of other single-neuron variables such as firing rate [5]. While the fact that correlated activity can significantly depend on stimulus statistics as well as the animal’s behavioral state [6–9] supports the hypothesis that correlated activity could code for behaviorally relevant stimulus features, this argument has been challenged by the fact that neural correlations have been shown to covary with firing rate [10,11].

On the other hand, natural sensory stimuli have rich spatiotemporal structure and are frequently characterized by time-varying moments such as mean and variance [12–18]. While psychophysical studies have shown that both stimulus and variance are critical for perception [16,17,19–22], how these attributes are coded for in the brain remain largely unknown in general.

While it is clear that single neurons can respond to envelopes when the stimulus intensity is high [23–28], a recent experimental study has instead focused on the coding of low-intensity stimuli [29]. Interestingly, Metzen *et al.* showed that, in this regime, correlated but not single-neuron activity could provide detailed information about envelope stimuli in both electroreceptors of weakly electric fish and vestibular afferents of Macaque monkeys. These results could moreover be reproduced by numerically simulating a pair of leaky integrate-and-fire neurons receiving common input and the coding of envelopes by correlated activity was further shown to be optimal for nonzero levels of variability. However, there as been no theoretical explanation of this phenomenon to date.

Here we provide a theoretical explanation of the phenomenon. We first introduce the model before deriving a relationship between the correlation coefficient and the stimulus amplitude. We then demonstrate that this theoretical expression is widely applicable and can accurately quantify results obtained while systematically varying model parameters. We then show that this theoretical expression can accurately describe time-varying changes in correlated activity in response to signals with time-varying amplitude.

## II. MODEL

**FIG. 1:**
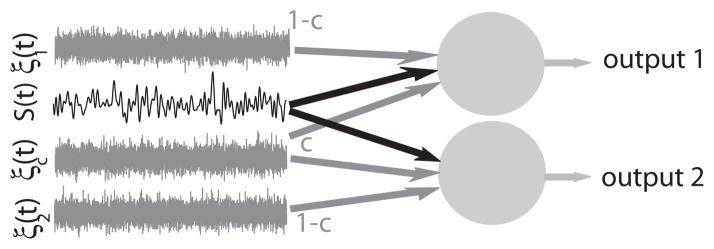
Model description. Model schematic in which two neurons receive a common time varying signal *S*(*t*), a common noise *ξ_c_*(*t*), and independent noise sources *ξ*_1_(*t*), *ξ*_2_(*t*). The parameter *c* controls the amount of correlated noise that the two neurons receive.

We consider two model neurons that receive a common signal *S*(*t*) as well as correlated *ξ_c_*(*t*) and uncorrelated noise sources *ξ*_1_(*t*) and *ξ*_2_(*t*) (Fig. 1).

(1) $${\dot{v}}_{1}=f(v_{1})+\mu_{1}+S(t)+\sqrt{1-c}\;\xi_{1}(t)+\sqrt{c}\;\xi_{c}(t),$$

(2) $${\dot{v}}_{2}=f(v_{2})+\mu_{2}+S(t)+\sqrt{1-c}\;\xi_{1}(t)+\sqrt{c}\;\xi_{c}(t),$$

where *v_j_* is the transmembrane voltage of neuron *j*, *μ_j_* is a bias current, and the parameter *c* controls the amount of correlated noise that both model neurons receive. Here *ξ*_1_(*t*), *ξ*_2_(*t*), and *ξ_c_*(*t*) are Gaussian white noise processes with zero mean and autocorrelation function 〈*ξ_i_*(*t*)*ξ_k_*(*t*′)〉 = 2*Dδ_ik_δ*(*t*−*t*′). The signal *s*(*t*) is taken to be low-pass filtered white noise with zero mean, variance *σ*^2^, and constant power up to a cut-off frequency *f_c_*. When *v_j_* is greater than a threshold *θ_j_*, a spike is said to have occurred and *v_j_* is then immediately reset to 0 and kept there for the duration of the absolute refractory period *T_j_*. We note that various forms for *f*(*V*) have been considered previously including the integrate-and-fire neuron [*f*(*V*) = 0] [30], the leaky integrate-and-fire neuron [*f*(*V*) = −*V*/*τ*] [31], and the exponential integrate-and-fire neuron (*f*(*V*) = Δexp [(*V* − *θ*)/Δ]) [32].

## III. THEORY

We quantified correlated neural activity by the cross-correlation coefficient *ρ*, which is defined by [6,10,11,29]:

(3) $$\rho =\frac{\int_{-\infty }^{\infty }d\tau \;\langle X_{1}(t)X_{2}(t+\tau )\rangle }{\sqrt{\int_{-\infty }^{\infty }d\tau \;\langle X_{1}(t)X_{1}(t+\tau )\rangle \int_{-\infty }^{\infty }d\tau \;\langle X_{2}(t)X_{2}(t+\tau )\rangle }},$$

where $X_{j}(t)=\sum_{i}\delta (t-t_{i}^{j})$ and { $t_{i}^{j}$} are the spike train and spike times of neuron *j*, respectively with *j* = 1,2. We also computed the cross-correlation function (i.e., the cross correlogram) between the spike trains *X*_1_(*t*) and *X*_2_(*t*) as:

(4) $$R(\tau )=\frac{\langle X_{1}(t)X_{2}(t+\tau )\rangle }{\sqrt{\langle X_{1}(t)X_{1}(t+\tau )\rangle \;\langle X_{2}(t)X_{2}(t+\tau )\rangle }},$$

where 〈 · 〉 denotes the average over realizations of the noises *ξ*_1_(*t*), *ξ*_2_(*t*), *ξ_c_*(*t*). We note that these are equivalent to averages over time due to stationarity.

Our goal is to derive an expression relating the cross-correlation coefficient *ρ* to the signal standard deviation *σ*, which is proportional to the envelope. Experimental and modeling results have shown that coding of envelopes by correlated but not single-neuron activity was observed only when the stimulus gave rise to linearly related modulations in firing rate [29]. Thus, we used linear response theory [33] and assumed that the perturbed spike train of neuron *j* is given by:

(5) $${\tilde{X}}_{j}(f)={\tilde{X}}_{0j}(f)+\chi_{j}(f)\tilde{S}(f),$$

where *X̃_j_*(*f*) is the Fourier transform of *X_j_*(*t*) and *X̃*_0_*_j_*(*f*) is the Fourier transform of the unperturbed (i.e., in the absence of stimulation) spike train, *X*_0_*_j_*(*t*), which we also assume to be stationary. Here *S̃*(*f*) is the Fourier transform of *S*(t), and *χ_j_*(*f*) is the susceptibility. Using Parseval’s theorem, we obtain that the correlation coefficient *ρ* is given by [11]:

(6) $$\rho =\frac{|P_{X1X2}(0)|}{\sqrt{P_{X1}(0)P_{X2}(0)}}=\sqrt{C(0)}.$$

*C*(*f*) is the coherence function given by:

(7) $$C(f)=\frac{{|P_{X1X2}(f)|}^{2}}{P_{X1}(f)P_{X2}(f)}.$$

Here *P_X_*_1_*_X_*_2_(*f*) is the cross spectrum between spike trains *X*_1_(*t*), *X*_2_(*t*), $P_{Xj}(f)=\langle {\tilde{X}}_{j}^{*}(f){\tilde{X}}_{j}(f)\rangle$ is the power spectrum of spike train *X_j_*(*t*). We have:

(8) $$P_{X1X2}(f)=\langle {\tilde{X}}_{1}^{*}(f){\tilde{X}}_{2}(f)\rangle ,$$

(9) $$P_{Xj}(f)=\langle {\tilde{X}}_{j}^{*}(f){\tilde{X}}_{j}(f)\rangle ,$$

where “^*^” denotes complex conjugation. Using Eq. (5), Eq. (8) becomes:

(10) $$P_{X1X2}(f)=[{\tilde{X}}_{01}^{*}(f)+\chi_{1}^{*}(f){\tilde{S}}^{*}(f)][{\tilde{X}}_{02}(f)+\chi_{2}(f)\tilde{S}(f)]$$

(11) $$=\langle {\tilde{X}}_{01}^{*}(f){\tilde{X}}_{02}(f)\rangle +\langle \chi_{1}^{*}(f)\chi_{2}(f)P_{S}(f)\rangle ,$$

where *P_S_*(*f*) = 〈S̃^*^(*f*)*S̃*(*f*)〉 is the stimulus power spectrum and were we have used $\langle {\tilde{X}}_{0j}^{*}(f)\tilde{S}(f)\rangle =0$, which follows trivially from the fact that the stimulus *S*(*t*) is by definition uncorrelated with the baseline activities of the model neurons *X*_0_*_j_*(*t*). Using Eq. (5) in Eq. (9) gives:

(12) $$P_{Xj}(f)=\langle [{\tilde{X}}_{0j}^{*}(f)+\chi_{j}^{*}(f){\tilde{S}}^{*}(f)]\times [{\tilde{X}}_{0j}(f)+\chi_{j}(f)\tilde{S}(f)]\rangle$$

(13) $$=P_{X0j}(f)+{|\chi_{j}(f)|}^{2}P_{S}(f),$$

where $P_{X0j}(f)=\langle {\tilde{X}}_{0j}^{*}(f){\tilde{X}}_{0j}(f)\rangle$ is the power spectrum of the baseline spike train *X*_0_*_j_*(*t*). We note that Eq. (13) is only valid in the limit of high noise [33].

Using Eqs. (11) and (13) in Eq. (6) gives:

(14) $$\rho =\frac{|\langle {\tilde{X}}_{01}^{*}(0){\tilde{X}}_{02}(0)\rangle |+|\chi_{1}(0)|\;|\chi_{2}(0)|P_{S}(0)}{\sqrt{\prod_{j=1}^{2}\left[P_{0j}(0)+{|\chi_{j}(0)|}^{2}P_{S}(0)\right]}},$$

where we have again used $\langle {\tilde{X}}_{0j}^{*}(f)\tilde{S}(f)\rangle =0$. Now, we use the fact that, for a linear system, the correlation coefficient between the outputs *X*_01_(*f*), *X*_02_(*t*) is equal to the correlation coefficient between the input noises *c* [10] to obtain:

(15) $$|\langle {\tilde{X}}_{01}^{*}(f){\tilde{X}}_{02}(f)\rangle |=c\sqrt{P_{X01}(f)P_{X02}(f)}.$$

Moreover, we have the following expressions for the power spectra at zero frequency [34,35]:

(16) $$P_{0j}(0)=\nu_{0j}CV_{0j}^{2}\sum_{i=-\infty }^{+\infty }\kappa_{ji}$$

(17) $$P_{S}(0)=\frac{\sigma^{2}}{2f_{c}},$$

where *ν_j_* is the baseline firing rate of neuron *j*, *CV*_0_*_j_* is the coefficient of variation defined as the standard deviation to mean ratio of the interspike interval distribution function, and *κ_ji_* is the *i*th correlation coefficient of the interspike interval sequence ${\left\{I_{i}^{j}\right\}}_{i=1}^{N}$ with $I_{i}^{j}=t_{i+1}^{j}-t_{i}^{j}$ of neuron *j*:

(18) $$\kappa_{ij}=\frac{{\langle (I_{k+i}^{j}-{\langle I_{k}^{j}\rangle }_{k})(I_{k}^{j}-{\langle I_{k}^{j}\rangle }_{k})\rangle }_{k}}{{\langle {\left(I_{k}^{j}-{\langle I_{k}^{j}\rangle }_{k}\right)}^{2}\;\;\;\mathrm{big}\rangle }_{k}},$$

where ${\langle \ldots \rangle }_{k}=\frac{1}{N}\sum_{k=1}^{N}\ldots$ and we have assumed that the interspike interval sequence ${\left\{I_{i}^{j}\right\}}_{i=1}^{N}$ is stationary.

Finally, using Eqs. (15)–(17) in Eq. (14), we obtain:

(19) $$\rho =\frac{c\prod_{j=1}^{2}\left[\sqrt{\nu_{0j}}CV_{0j}\sqrt{\sum_{i=-\infty }^{+\infty }\kappa_{ji}}\right]+|\chi_{1}(0)\chi_{2}(0)|\frac{\sigma^{2}}{2f_{c}}}{\sqrt{\prod_{j=1}^{2}\left[\nu_{0j}CV_{0j}^{2}\sum_{i=-\infty }^{+\infty }\kappa_{ji}+{|\chi_{j}(0)|}^{2}\frac{\sigma^{2}}{2f_{c}}\right]}}.$$

We note that Eq. (19) can be readily evaluated as analytical expressions for the susceptibilities *χ_j_* (0), mean firing rates *ν*_0_*_j_*, and coefficient of variations *CV*_0_*_j_* have been obtained elsewhere for several integrate-and-fire type models [36,37].

We note that, if we assume homogeneity, then we have *ν*_0_ = *ν*_01_ = *ν*_02_, *CV*_0_ = *CV*_01_ = *CV*_02_, *χ*(0) = *χ*_1_(0) = *χ*_2_(0), and *κ_i_* = *κ*_1_*_i_* = *κ*_2_*_i_*. Eq. (19) then simplifies to:

(20) $$\rho =\frac{1+\frac{2cf_{c}\nu_{0}CV_{0}^{2}\sum_{i=-\infty }^{+\infty }\kappa_{i}}{{|\chi (0)|}^{2}\sigma^{2}}}{1+\frac{2f_{c}\nu_{0}CV_{0}^{2}\sum_{i=-\infty }^{+\infty }\kappa_{i}}{{|\chi^{\left(0\right)}|}^{2}\sigma^{2}}}.$$

For a renewal process [38], we have [35]:

(21) $$\sum_{i=-\infty }^{+\infty }\kappa_{ji}=1$$

and Eq. (20) further simplifies to:

(22) $$\rho =\frac{1+2cf_{c}\nu_{0}CV_{0}^{2}{|\chi^{\left(0\right)}|}^{-2}\sigma^{-2}}{1+2f_{c}v_{0}CV_{0}^{2}{|\chi (0)|}^{-2}\sigma^{-2}}.$$

## IV. RESULTS

In the following, we will assume *f*(*v*) = 0 (i.e., perfect integrate-and-fire dynamics) or *f*(*v*) = −*v* (i.e., leaky integrate-and-fire) for numerical results. Further, if we assume a negligible absolute refractory period (i.e., *T* = *T*_1_ = *T*_2_ = 0), then we have $CV_{0}=\sqrt{1-\exp\;{\left[-\mu \theta /(2D)\right]}^{2}}/(1-\exp\;[-\mu \theta /(2D)])$, *ν*_0_ = *μ*/*θ*,, $\chi (0)=\frac{1}{\theta }$ for the perfect integrate-and-fire neuron [36,37], which allows calculation of the correlation coefficient *ρ* as a function of model parameters directly through Eq. (22).

### A. Dependence on stimulus amplitude

**FIG. 2:**
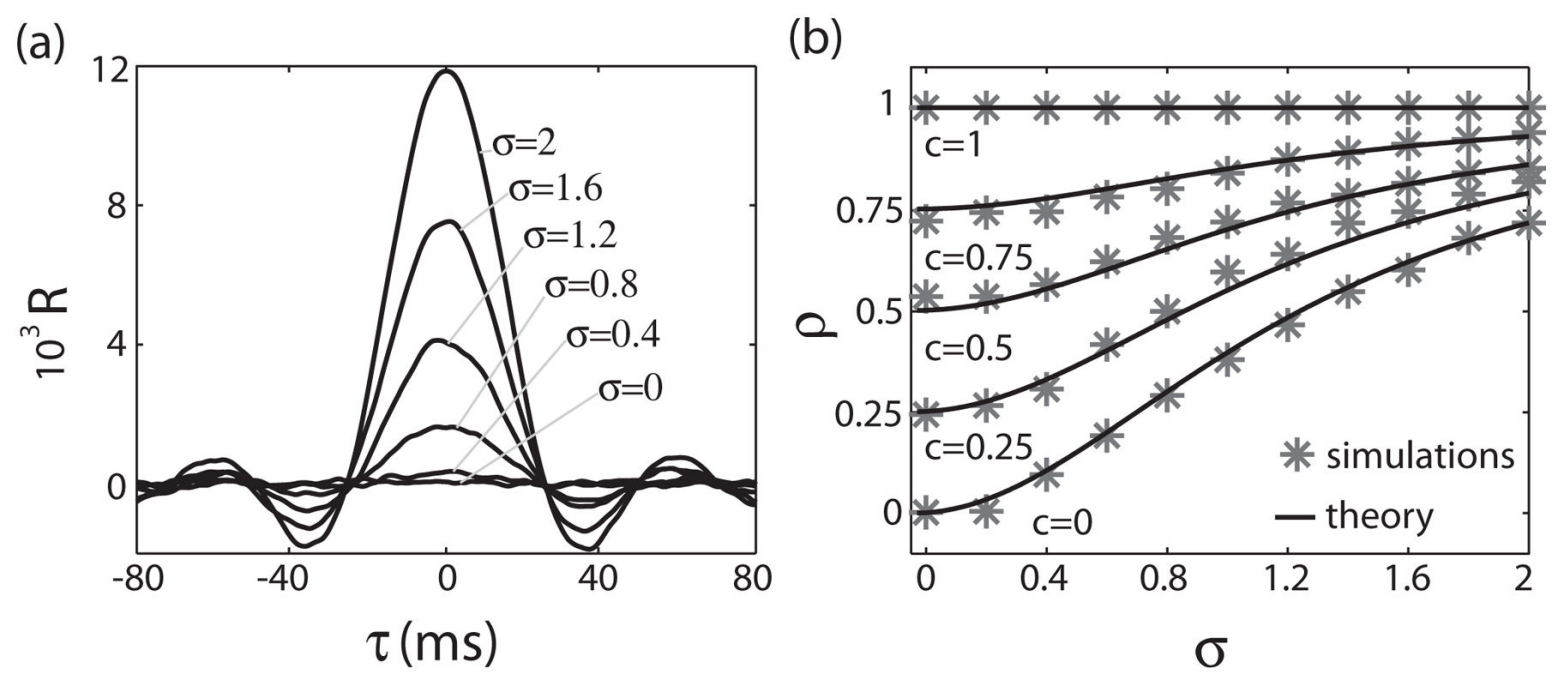
Correlated activity is a function of stimulus amplitude for the perfect integrate-and-fire neuron model. (a) Cross-correlation function *R* as a function of *τ* for different values of *σ*. We used *μ* = 4, *T* = 0, *θ* = 11, *D* = 500, *f_c_* = 20. (b) *ρ* as a function of *σ* for different values of *c* for numerical simulations (asterisks) and from Eq. (22) (solid line). Other parameters are the same as in (a).

We first focus on how the cross-correlation function *R* varies with stimulus amplitude *σ* when the noise sources are uncorrelated (i.e., *c* = 0). Figure 2(a) shows *R* as a function of *τ* for several values of *σ*. It is seen that the cross-correlation function increases near *τ* = 0 with increasing *σ*. Further, the area under the curve also increases with increasing *σ*. This is reflected in the fact that the cross-correlation coefficient *ρ* increases as a function of *σ* [Fig. 2(b)]. We found that the values of *ρ* computed from numerical simulation are in excellent agreement with those predicted from Eq. (22).

### B. Dependence on fraction of common noise *c*

Interestingly, increasing the fraction of correlated noise input *c* limits the range of values that *ρ* can take as we have lim*_σ_*_→ 0_ *ρ* = *c*. Therefore, we have *c* ≤ *ρ* ≤ 1 and increasing *c* is thus detrimental to variance coding by correlated activity. Indeed, in the limit where noise inputs are perfectly correlated (i.e., *c* = 1), it can be seen that *ρ* ≡ 1 independently of *σ* by inspecting Eq. (22). This result is confirmed by our numerical simulations, which are in excellent agreement with theoretical predictions [Fig. 2(b)].

### C. Dependence on other parameters

**FIG. 3:**
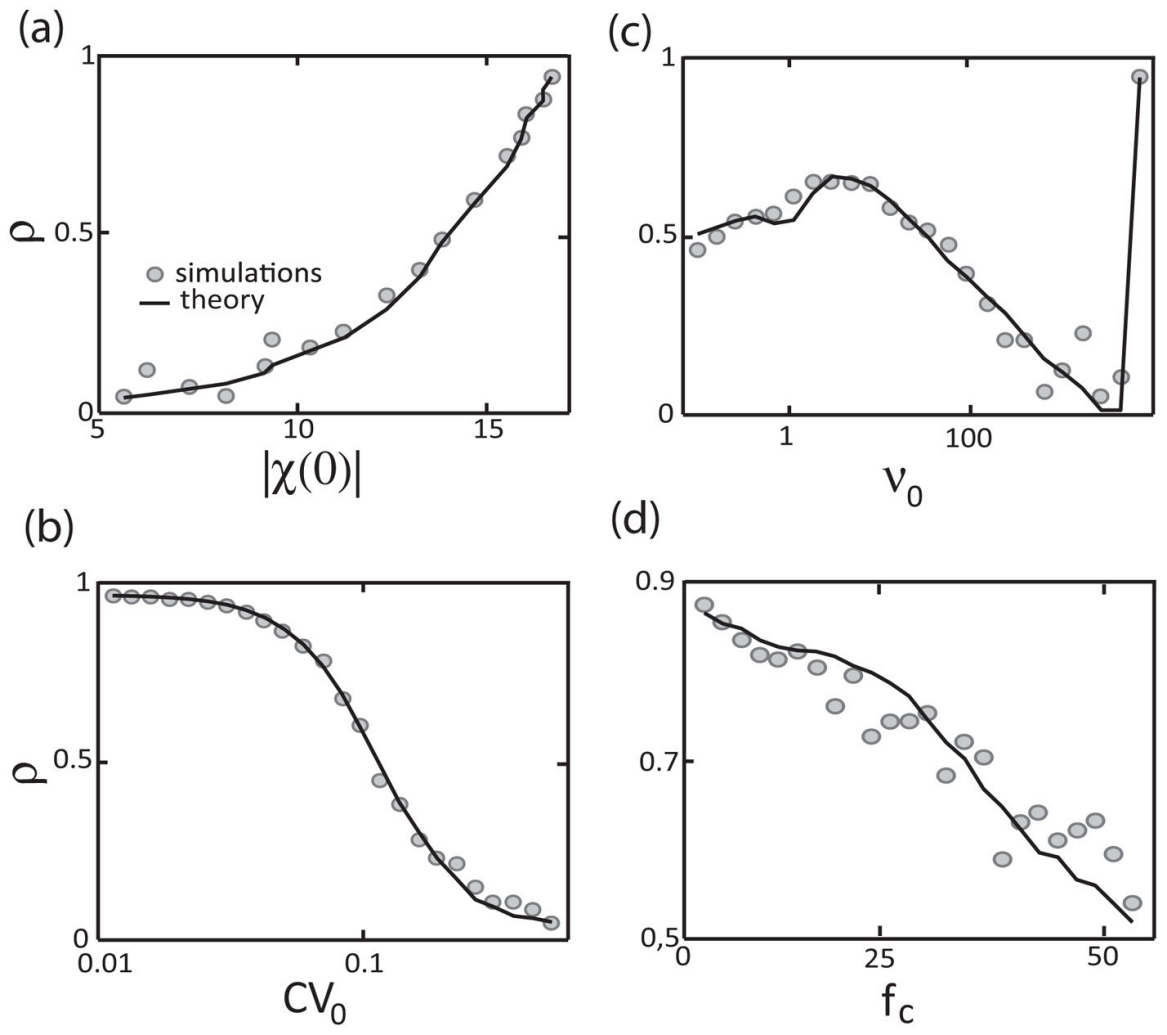
Influence of |*χ*(0)|, *CV*_0_, *ν*_0_, and *f_c_* on correlation coding. (a) Correlation coefficient *ρ* as a function of |*χ*(0)|. (b) Correlation coefficient *ρ* as a function of *CV*_0_. (c) Correlation coefficient *ρ* as a function of *ν*_0_. (d) Correlation coefficient *ρ* as a function of *f_c_*. The gray circles show the values obtained from numerical simulations and the solid black line the values obtained from Eq. (20). The simulations were obtained from a leaky integrate-and-fire model using the same parameter values as in Ref. [29] by varying both the bias current and noise intensity.

We next tested whether our theoretical predictions would hold while systematically varying other model parameters. This is important because it is well known that calculations based on linear response theory only hold for weak stimulus intensities and large noise values [33,37,39,40]. We tested this by comparing values obtained for *ρ* from numerical simulations to those obtained from Eq. (20) for different values of |*χ*(0)| [Fig. 3(a)], *CV*_0_ [Fig. 3(b)], *ν*_0_ [Fig. 3(c)], and *f_c_* [Fig. 3(d)]. Surprisingly, we found good agreement between both values despite the fact that some parameters varied by several orders of magnitude.

### D. Dependence on variability and noise intensity

In order to better characterize the dependence of *ρ* on *σ*, we considered the partial derivative $\frac{\partial \rho }{\partial \sigma }$, henceforth referred to as the correlation susceptibility *G*, which is given by:

(23) $$G=\frac{\partial \rho }{\partial \sigma }=\frac{4\sigma (1-c)\;CV_{0}^{2}f_{c}\nu_{0}}{{|\chi^{\left(0\right)}|}^{2}{\left[\sigma^{2}+2|\chi {\left(0\right)}^{-2}|CV_{0}^{2}f_{c}\nu_{0}\right]}^{2}}.$$

It is easily seen that *G* ≥ 0 and thus that *ρ* is an increasing function of *σ*. Further, examining Eq. (23), we find that, for 0 ≤ *c* < 1 and *σ* > 0, *G* must display a maximum when *CV*_0_ is varied and when all other parameters are fixed because we then have *G* > 0 and lim_*CV*_0_→0_ *G* = lim_*CV*_0_→∞_ *G* = 0. Indeed, *G* is maximum for a nonzero value of *CV*_0_, *CV*_opt_, which is given by:

(24) $$CV_{\text{opt}}=\sigma \sqrt{\frac{3{|\chi (0)|}^{2}}{2fc\;v_{0}}}.$$

**FIG. 4:**
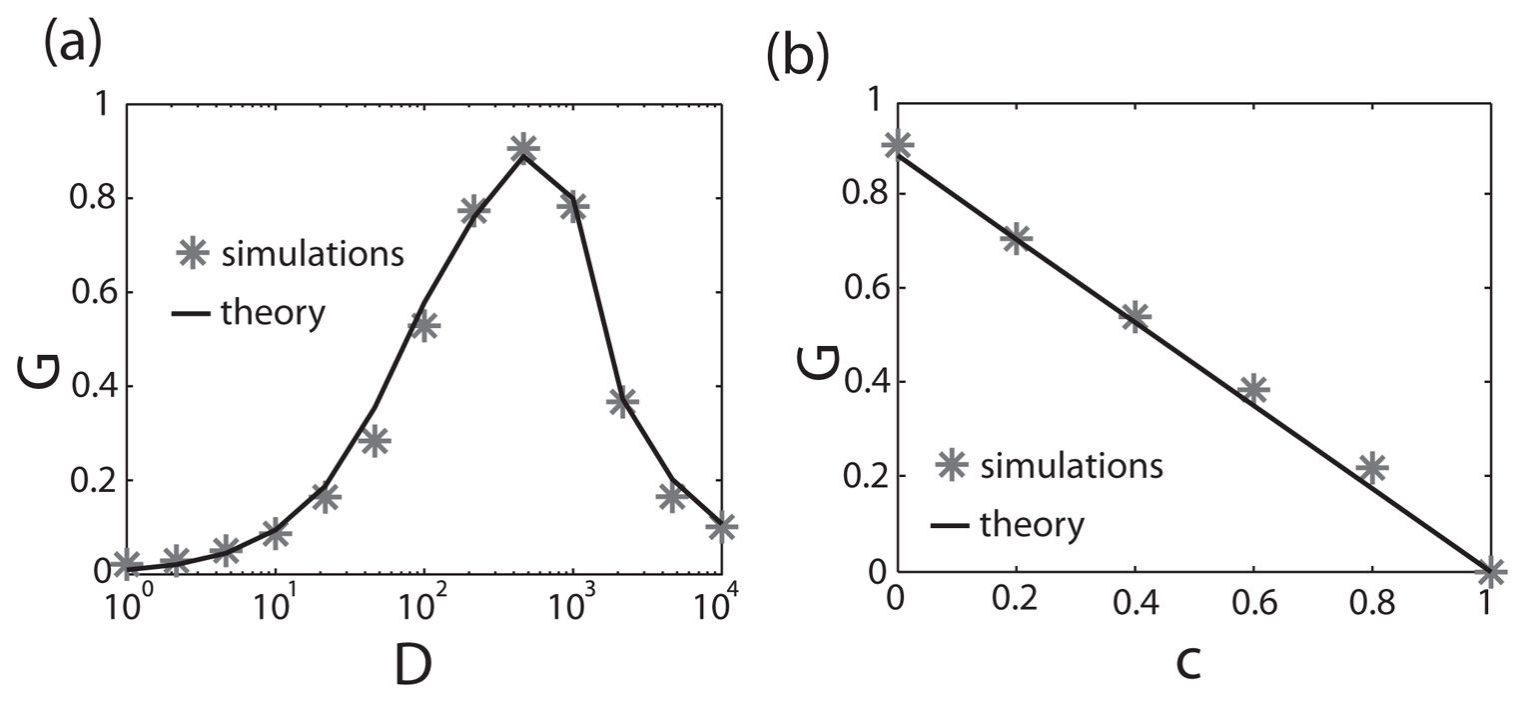
Influence of *D* and *c* on correlation coding (a) Correlation gain *G* as a function of *D* with the “*” showing numerical simulations and the solid black line is the prediction from Eq. (23). (b) *G* as a function of *c* with the “*” showing numerical simulations and the solid black line is the prediction from Eq. (23) Parameter values are the same as in Fig. 2(a) with *σ* = 1.

Since *CV*_0_ is a monotonously increasing function of noise intensity *D* [37], this implies that there is an optimal value of the noise intensity *D* for which the correlation susceptibility *G* is maximum. This prediction is verified and in excellent agreement with numerical simulations [Fig. 4(a)] using a perfect integrate-and-fire model for which we have [36,37]:

(25) $$CV_{0}=\frac{\sqrt{1-\exp\;{\left[-\mu \theta /(2D)\right]}^{2}}}{1-\exp\;[-\mu \theta /(2D)]}.$$

Further, Eq. (23) predicts that the correlation susceptibility *G* will decrease linearly as a function of the amount of correlated noise *c* and reaches 0 when *c* = 1. Numerical simulations are in excellent agreement with this prediction [Fig. 4(b)].

### E. Coding of time varying amplitude

**FIG. 5:**
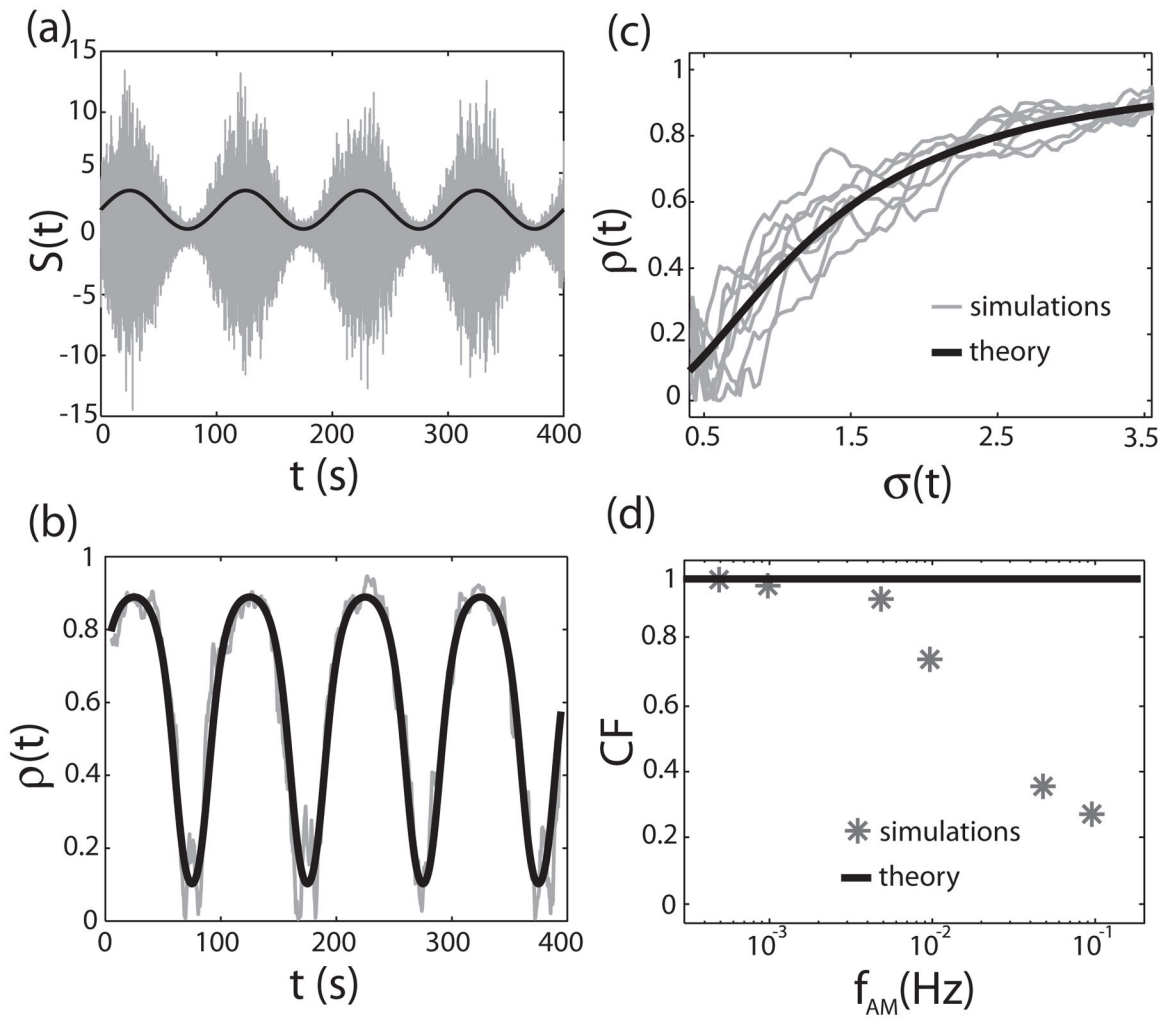
(a) amplitude-modulated noise signal *S*(*t*) (gray) whose standard deviation, *σ*(*t*) (black), varies sinusoidally in time. We used *A*_0_ = 0.8, *f_c_* = 10, and *f_AM_* = 0.0005, *σ*_0_ = 2. (b) *ρ*(*t*) from numerical simulations (gray) and from Eq. (22) (black) as a function of time. Other parameters have the same values as in Fig. 2(a). (c) *ρ*(*t*) versus *σ*(*t*) from numerical simulations (gray) and from theory (black). (d) Coding fraction *CF* versus *f_AM_* from numerical simulations (asterisks) and from theory (black line). We used a perfect integrate-and-fire model. Other parameter values were the same as in Fig. 2(a).

We next explore whether the correlation coefficient *ρ* can code for stimuli whose amplitude *σ* varies in time. To do so, we consider signals *s*(*t*), which consist of low-pass filtered Gaussian white noise (cut-off frequency *f_c_*) with zero mean and standard deviation *σ*(*t*) = *σ*_0_ [1 + *A*_0_ sin(2*πf_AM_t*)]. An example of such a signal is shown in Fig. 5(a). Using a quasistatic approximation, which assumes that *f_AM_* ≪ *f_c_*, Eq. (22) becomes:

(26) $$\rho (t)=\frac{1+2cf_{c}\nu_{0}CV_{0}^{2}{|\chi (0)|}^{-2}/\sigma^{2}(t)}{1+2f_{c}\nu_{0}CV_{0}^{2}{|\chi (0)|}^{-2}/\sigma^{2}(t)}.$$

In practice, we estimated the correlation coefficient *ρ*(*t*) from finite time windows with length $T_{\text{win}}=\frac{1}{10\;f_{AM}}$. Our numerical simulations show that the correlation coefficient *ρ*(*t*) does indeed follow the time course of *σ*(*t*) [Fig. 5(b)] and that there is a one-to-one relationship between the two [Fig. 5(c)] similar to experimental data [29]. Importantly, the time-varying correlation coefficient *ρ*(*t*) computed using Eq. (26) was in excellent agreement with that computed from numerical simulations [Figs. 5(b) and 5(c)].

We next explored how *f_AM_* influences variance coding by correlations. To do so, we computed the coding fraction *CF* defined by $CF=1-\frac{{\langle {\left[\rho_{\text{est}}(t)-\rho (t)\right]}^{2}\rangle }_{t}}{{\langle \rho {\left(t\right)}^{2}\rangle }_{t}}$, where 〈…〉*_t_* denotes the average over time *t*, *ρ*_est_(*t*) is the estimated correlation coefficient and *ρ*(*t*) is the theoretical prediction obtained from Eq. (22). We note that, in theory, we should get *CF* = 1. Our numerical simulations show that this is indeed the case for low values of *f_AM_* [Fig. 5(d)] but that *CF* drops for higher values of *f_AM_*, which is expected as the quasi-static approximation does not hold anymore. Correlation coding by correlations is thus most effective when the signal amplitude *σ*(*t*) varies slowly with respect to the signal *s*(*t*) itself.

## V. DISCUSSION

In summary, we have investigated how correlated neural activity can code for signal variance. We found that such coding was optimal when no noise correlations were present (i.e., *c* = 0) and for a nonzero value of noise intensity *D*. Finally, we have shown that correlated activity could code for signals whose variance varies in time and that such coding was best when the variance varies slowly in time. We note that, although our results were obtained for the perfect integrate-and-fire neurons, they hold in other variants of this model such as the leaky and exponential versions (data not shown). Further, these results also hold in the Hodgkin-Huxley model as verified by numerical simulations (data not shown). It should also be noted that the expressions obtained for the correlation coefficient as a function of stimulus variance, namely Eqs. (19), (20), and (22), are generic and are applicable to any neuron model provided that the assumptions for linear response theory are valid.

Our results show that correlation coding of stimulus amplitude is best for stimuli whose variance varies slowly in time with respect to other moments. This is not expected to be an issue as this is the case for most natural stimuli [17,41,42]. Further, our results provide a new functional role for neuronal variability. It is well known that neurons in the brain display trial-to-trial variability in their responses to repeated presentations of the same stimulus and there is still much debate as to the role played by this variability in coding [43] as well as its origins [44]. What is most surprising is that, in some systems, neurons that display low variability coexist with neurons that display high variability. This is the case in the peripheral vestibular system where there are two classes of afferents: one displays very little trial-to-trial variability whereas the other displays high trial-to-trial variability [45,46]. We predict that the latter class will be more suited towards the coding of stimulus variance by neural correlations.

Our results using linear response theory assume that the signal *s*(*t*) introduces only a small perturbation to the unperturbed activity *X*_0_(*t*) [33]. It is thus clear that amplitude coding by neural correlations will not occur in every neuron within the brain. Rather, we predict that such coding will tend to occur mostly in neurons that display a high firing rate in the absence of stimulation and that do not share synaptic connections with other neurons, thereby minimizing sources of correlated noise. This seems to be the case for peripheral sensory neurons. For example, electroreceptor afferents in weakly electric are characterized by high baseline (i.e., in the absence of stimulation) firing rates and no noise correlations [47]. However, other peripheral as well as central sensory neurons are also characterized by high baseline firing rates [45,46,48–51]. While it is clear that many central neurons receive correlated noise [2], our results suggest that they would not hinder variance coding by correlations at the levels reported in experiments. It is thus likely that variance coding by correlations will be found across the brain.

We note that information about stimulus variance is not carried by the single-neuron firing rate as it is constant over the timescale over which the variance varies. This of course assumes that the model neuron activity is well described by linear response theory. In the case where significant nonlinearities are present, information about stimulus variance can also be carried by single-neuron attributes such as firing rate [5,23–29]. In this case, as the single-neural firing rate and correlations covary [10,11], there is redundancy between the information carried by the firing rate and correlations. Further, such nonlinearities can introduce ambiguity in the neural code as they can then code for both stimulus mean and variance [52]. We propose that correlated activity can code for stimulus amplitude in parallel while single-neuron attributes such as firing rate or spike timing can instead code for stimulus mean. Both sources of information can then be decoded by downstream neurons using different mechanisms and thereby segregate both information streams as is observed experimentally [27]. In particular, while it is unlikely that the brain actually computes a correlation coefficient, previous experimental work has shown that realistic neural circuits can successfully decode information carried by correlated neural activity [29].
